# Cost of start-up activities to implement a community-level opioid overdose reduction intervention in the HEALing Communities Study

**DOI:** 10.1186/s13722-024-00454-w

**Published:** 2024-04-02

**Authors:** Iván D. Montoya, Colleen Watson, Arnie Aldridge, Danielle Ryan, Sean M. Murphy, Brenda Amuchi, Kathryn E. McCollister, Bruce R. Schackman, Joshua L. Bush, Drew Speer, Kristin Harlow, Stephen Orme, Gary A. Zarkin, Mathieu Castry, Eric E. Seiber, Joshua A. Barocas, Benjamin P. Linas, Laura E. Starbird

**Affiliations:** 1 Department of Public Health Sciences, University of Miami Miller School of Medicine, Miami, FL USA; 2 RTI International, Research Triangle Park, NC USA; 3 Department of Population Health Sciences, Weill Cornell Medical College, New York, NY USA; 4 Section of General Internal Medicine, Boston University School of Medicine, Boston, MA USA; 5 College of Public Health, University of Kentucky, Lexington, KY USA; 6 College of Public Health, The Ohio State University, Columbus, OH USA; 7 Sections of General Internal Medicine and Infectious Diseases, University of Colorado Anschutz Medical Campus, Aurora, CO USA; 8 Department of Family and Community Health, University of Pennsylvania School of Nursing, Philadelphia, PA USA

**Keywords:** Community engagement, Cost analysis, Opioid use disorder, Start-up cost, Intervention implementation

## Abstract

**Background:**

Communities That HEAL (CTH) is a novel, data-driven community-engaged intervention designed to reduce opioid overdose deaths by increasing community engagement, adoption of an integrated set of evidence-based practices, and delivering a communications campaign across healthcare, behavioral-health, criminal-legal, and other community-based settings. The implementation of such a complex initiative requires up-front investments of time and other expenditures (i.e., start-up costs). Despite the importance of these start-up costs in investment decisions to stakeholders, they are typically excluded from cost-effectiveness analyses. The objective of this study is to report a detailed analysis of CTH start-up costs pre-intervention implementation and to describe the relevance of these data for stakeholders to determine implementation feasibility.

**Methods:**

This study is guided by the community perspective, reflecting the investments that a real-world community would need to incur to implement the CTH intervention. We adopted an activity-based costing approach, in which resources related to hiring, training, purchasing, and community dashboard creation were identified through macro- and micro-costing techniques from 34 communities with high rates of fatal opioid overdoses, across four states—Kentucky, Massachusetts, New York, and Ohio. Resources were identified and assigned a unit cost using administrative and semi-structured-interview data. All cost estimates were reported in 2019 dollars.

**Results:**

State-level average and median start-up cost (representing 8–10 communities per state) were $268,657 and $175,683, respectively. Hiring and training represented 40%, equipment and infrastructure costs represented 24%, and dashboard creation represented 36% of the total average start-up cost. Comparatively, hiring and training represented 49%, purchasing costs represented 18%, and dashboard creation represented 34% of the total median start-up cost.

**Conclusion:**

We identified three distinct CTH hiring models that affected start-up costs: hospital-academic (Massachusetts), university-academic (Kentucky and Ohio), and community-leveraged (New York). Hiring, training, and purchasing start-up costs were lowest in New York due to existing local infrastructure. Community-based implementation similar to the New York model may have lower start-up costs due to leveraging of existing infrastructure, relationships, and support from local health departments.

**Supplementary Information:**

The online version contains supplementary material available at 10.1186/s13722-024-00454-w.

## Background

The opioid overdose crisis is one of the most pressing public health issues in the United States. Community-based opioid overdose education and naloxone distribution (OEND) programs, medications for opioid use disorder (MOUD), and safer prescribing and dispensing education are effective public health interventions to prevent opioid-related overdose [11, 14, 18]. Despite the demonstrated efficacy of these evidence-based practices (EBPs) to support harm reduction, treatment, and recovery from opioid use disorder (OUD), there are substantial barriers to implementing them. Fewer than 20% of people with OUD receive recommended treatment and services [19]. Reasons for underutilization of these services include a limited number of MOUD prescribing providers, lack of screening for OUD by healthcare and justice systems, lack of treatment capacity for MOUD, lack of access and awareness among individuals with OUD about treatment options, and stigma surrounding the use of MOUD [3, 9, 10, 19].

Communities That Heal (CTH) is a community-engaged intervention designed to reduce opioid overdose fatalities by increasing the adoption and delivery of EBPs and reducing stigma in healthcare, behavioral health, criminal justice, and other key settings [9]. The CTH intervention relies on community engagement to assist key stakeholders in using data-driven techniques to select and implement EBPs, and a communication campaign to educate the community, address stigma, and build demand for EBPs. In the CTH model, communities establish a coalition of stakeholders from sectors including medical and mental health services, substance use treatment and harm reduction services, law enforcement and corrections, education, social services, local government, and individuals with lived experience. Guided by their community’s opioid-related data (e.g., overdose trends, hotspot mapping, law enforcement activity), each coalition selects strategies from a menu of EBPs targeting OEND, MOUD, and safer prescribing, and guides the implementation of these strategies. In addition, community coalitions deploy a series of communications campaigns to reduce stigma and increase uptake of MOUD and OEND. The HEALing Communities Study (HCS) is a multi-site, parallel group, cluster randomized, wait-list controlled trial to implement and evaluate the effect of the CTH intervention on reducing opioid overdose deaths in disproportionately affected communities located in four states– Kentucky, Massachusetts, New York, and Ohio. The goal of the HCS is to produce generalizable information for policy makers and community stakeholders seeking to implement CTH or similar community-driven interventions [1].

Implementing CTH requires communities to invest substantial time and resources in establishing a community-driven process for EBP selection and implementation. In a large-scale community intervention framework such as CTH, assessing these initial investments is critical and more complex than in a traditional individual-level randomized trial. This paper therefore describes the economic costs of the start-up phase of CTH that encompasses the activities required to begin implementing the intervention in the 34 communities randomized to initiate CTH in the first wave of the wait-list controlled HCS study. While recent economic evaluations have demonstrated the value of pharmacologic interventions for OUD [4–6, 17], start-up costs are frequently excluded from these economic evaluations. Few studies have reported start-up costs related to OEND and MOUD implementation [2, 7] and to our knowledge, this is the first study to report the initial investment required to implement a complex, large-scale, community-driven approach to addressing the opioid crisis in the United States.

## Methods

### Overview

We defined, measured, and valued the costs of start-up investments in four HCS study sites located in Kentucky, Massachusetts, New York, and Ohio. A total of 34 rural and urban intervention communities (counties, townships, or metropolitan areas) across these four sites, 8 communities in 3 states and 10 in Ohio, were randomized to implement the CTH intervention. We identified three different models of operating across the four sites: hospital-academic (Massachusetts), university-academic (Kentucky and Ohio), and community-leveraged (New York). The hospital-academic and university-academic models primarily hired staff through one institution while also taking advantage of the expertise of existing academic faculty. The community-leveraged model primarily hired or used existing staff at local government or community-based organizations, while also taking advantage of the expertise of faculty and staff located at an academic institution.

We estimated costs from the community perspective, reflecting the investments that a community would need to incur during preparation to implement the CTH intervention. In this case, community may be defined as a local or county government, health department, health system, or community-based organization; 24 of the communities in the HCS study represented counties and the other 10 represented units smaller than counties.

We adopted an activity-based costing approach in which the activities and resources to implement the start-up phase were first identified and then assigned unit costs. We defined start-up costs as all one-time, preparatory expenses incurred from inception of the CTH design until the CTH community coalitions were formed and functioning. We included all relevant costs that were incurred during the HCS trial preparation phase from May 2019 through December 2019, as well as costs incurred for start-up activities that occurred during the early intervention phase through April 2020. All costs are reported in 2019 U.S. dollars. Costs associated with implementing the CTH intervention will be presented in future analyses. HCS research costs (e.g., data collection and IRB compliance training, and staff hired to support research operations) during these time periods were excluded, which is consistent with our goal of understanding the resources needed to reproduce the intervention start-up outside of a research environment.

We identified four start-up cost categories: hiring intervention staff; training intervention staff; equipment and infrastructure; and costs to develop community online dashboards. We used a standardized instrument to systematically collect data across the four HCS sites for each of these categories.

All methods were carried out in accordance with the protocol and guidelines established by the Healing Communities Steering Committee and by Advarra Inc., the HEALing Communities Study single Institutional Review Board (IRB). Furthermore, the start-up cost analysis plan and methodology were developed by the Health Economics Workgroup (HEWG), which includes health economist members from the 4 study sites. Cost data collection was carried out through a standardized process agreed upon by all HEWG members. Policies and procedures to conduct semi-structured interviews with administrative staff were approved by the study IRB. In order to participate in the semi-structured interviews, staff received a verbal informed consent description and had to agree before the interview could commence. Staff informed consent was documented in REDCap. All subjects agreed to participate and provided consent.

### Hiring costs

Hiring costs include time invested by individuals involved in hiring intervention staff including human resources personnel, legal personnel, project directors, faculty (excluding time spent on research exclusive hiring activities), and community-level staff. The process of hiring staff varied across the sites. In Kentucky and Ohio, CTH intervention staff were hired as university employees. We conducted semi-structured interviews with the administrative staff who performed hiring activities at each university to understand the time they spent hiring CTH intervention staff beginning in May 2019 through December 2019. Informed consent was obtained from administrative staff and other respondents before collecting interview data. Interviewers walked interviewees through a standardized form and asked questions that would allow completion of the form (included in Additional file 1: Material). In Massachusetts, the intervention staff members were hired as hospital and university employees between July 2019 and December 2019, and hiring data were collected in interviews with relevant staff. In New York, intervention staff were hired by each CTH community’s local health department or lead community-based organization between October 2019 and April 2020, and time estimates for the hiring process were obtained individually from administrative staff in each of the communities. We recorded time spent by each hiring staff member on pre-hire activities, such as creating and posting job descriptions, reviewing applicants, interviewing applicants, hiring decision-making, and general onboarding activities (not including CTH-specific trainings, which were captured separately as training costs). An average per-hire time commitment was then calculated and applied to every intervention staff member hired.

The labor costs associated with these time estimates were calculated using salaries and fringe benefits that were obtained from site invoices, self-report and publicly available data sources. In a real-world scenario, CTH staff would likely not be hired by academic centers. Therefore, to account for the likelihood that community-level staff would be responsible for hiring, we replaced the actual academic researcher wages with wages based on comparable community occupations (i.e., Medical and Health Service Managers, Payroll and Timekeeping Clerks, across all sites and applied a nationally representative 34% fringe rate [16].

### Training costs

In Kentucky and Ohio, faculty and staff coordinated a central training for new intervention staff at the beginning of the intervention implementation period (i.e., post-December 2019). The original training was conducted in-person, but it was also recorded for viewing by subsequent hires. Training sessions in Massachusetts were conducted both in-person and virtually and were recorded and saved for future hires to view. In New York, both live and virtual training sessions were conducted, and each new hire completed a pre-recorded series of training modules.

The cost of training staff was valued using two time components: (1) time spent by individuals performing the training, and (2) time spent by intervention staff being trained. Time spent by trainers preparing materials and pre-recording trainings was estimated in interviews. Time spent by trainers and trainees in live and virtual sessions was estimated using study records detailing the training sessions at each site. Similar to calculating hiring costs, the labor costs associated with these time estimates were calculated using salaries and fringe benefits that were obtained from site invoices and human resources reports. To account for real-world implementation where academic faculty likely would not lead the intervention, we replaced the actual academic researcher wages with wages based on comparable community-based roles (i.e., Medical and Health Service Managers, Mental Health and Substance Abuse Social Workers, and Health Education Specialists) across all sites and applied a 34% fringe rate. In the few instances of staff turnover, start-up hiring cost estimates were limited to one instance per position filled.

### Equipment and infrastructure costs

The costs of physical space, information technology (IT) services, and equipment needed to prepare for CTH implementation were gathered from invoices, purchasing records, and interviews. The Kentucky site hired staff who lived in or near each intervention community and placed them in space at local universities, public health departments, or other suitable office locations. We recorded start-up costs associated with obtaining this space, IT infrastructure (e.g., access to wireless internet on the University of Kentucky network), and teleconferencing equipment for virtual interaction. The Ohio and Massachusetts sites reported equipment purchases, such as phones and laptops for intervention staff, but no new space or IT infrastructure costs. The New York site reported software costs and equipment costs such as phones, laptops, and desks. The New York site also did not have added space or IT infrastructure costs because the intervention staff were housed primarily in existing county health department and local partner organization offices.

### Community dashboard portal costs

The CTH intervention includes a community dashboard portal for sites to view and share community-specific data with their community stakeholders, and for stakeholders to use to inform community-level decisions about which EBPs to implement. The cost of establishing the portals was derived from time spent by CTH staff to help design the portal, time spent by computer programmers to create the portal, and in some cases, the cost of new software to create and/or host the portal. Costs associated with the time estimates were calculated using salaries and fringe benefits that were obtained from site human resource records and self-report.

### Analysis

We entered, cleaned, and analyzed data using a standardized MS-Excel spreadsheet. This data collection tool was standardized but flexible enough for sites to tailor data collection to their specific needs and included separate worksheets for each of the start-up cost categories (hiring, training, infrastructure and equipment, and dashboard costs). All sites had salary and fringe benefit information available for current staff and staff who were hired for the CTH intervention. In addition to using administrative data on hiring and training costs from each site, we also obtained standardized wage data from the Occupational Information Network (O*NET) website and applied a 34% fringe rate in order to compare the local site costs to regional and national costs for similar positions [15]. O*NET is a comprehensive database containing occupational characteristics, wages, and other information obtained through national surveys of sampled workers, occupation experts, and occupation analysts. O*NET wages were assigned by matching the average state and national salaries of occupational titles with similar job titles and duties as the CTH intervention staff (Additional file 1: Table S2).

We calculated start-up cost per capita for each state using the total population of Wave 1 HEALing Communities Study communities. Total community population represents the societal pool that would be responsible for the cost of the CTH intervention. The 24 county population estimates were retrieved from the 2020 Bridged-Race Population Estimates [12] and for the 10 communities that represent units smaller than counties, we used population estimates from the 2017–2021 American Community Survey 5-Year Averages [17].

## Results

Table 1 summarizes the cost of each of the sites’ start-up cost to prepare to implement the CTH intervention by each of the four cost categories (hiring, training, infrastructure and equipment, and dashboard costs). Across the four sites, the total state-level mean and median start-up costs were $247,673 and $175,683, respectively (range $149,776 to $358,404). The population was 786,387 in Kentucky, 451,629 in Massachusetts, 1,382,518 in New York, and 3,006,020 in Ohio. The resulting start-up cost per capita was $0.46 in Kentucky, $0.67 in Massachusetts, $0.11 in New York, and $0.06 in Ohio. Hiring and training represent 40% of the average total start-up cost. Infrastructure and equipment represent 24% of the average total start-up cost but 18% of the median cost; this difference is attributable to higher costs in Kentucky, due to investments in infrastructure for new space in participating communities. Dashboard costs represented 36% of average total startup costs but varied widely among the sites, from 6% of start-up costs in Kentucky to 71% of start-up costs in Massachusetts.

**Table 1:** Start-up cost of implementing the communities that HEAL intervention across all sites

Cost Category	KY		MA		NY		OH		Mean		Median	
	Cost	%	Cost	%	Cost	%	Cost	%	Cost	%	Cost	%
Hiring	$112,602	31	$30,513	10	$9,487	6	$27,471	15	$45,018	18	$28,992	17
Training	$62,260	17	$41,212	14	$65,233	44	$48,461	27	$54,292	22	$55,361	32
Equipment/infrastructure	$161,888	45	$17,126	6	$14,845	10	$47,571	26	$60,357	24	$32,349	18
Dashboard	$21,654	6	$212,408	71	$60,211	40	$57,752	32	$88,006	36	$58,982	34
Total	$358,404	100	$301,259	100	$149,776	100%	$181,255	100	$247,673	100	$175,683	100
Total cost per capita	$0.46	–	$0.67	–	$0.11	–	$0.06	–	$0.32	–	$0.28	–

All costs reported in 2019 dollars. For communities that represent counties (n = 24 of 34), population estimates are from 2020 Bridged-Race Population Estimates retrieved via https://www.cdc.gov/nchs/nvss/bridged_race.htm on November 2, 2023. For communities that represent units smaller than counties (n = 10 of 34), population estimates are from 2017–2021 American Community Survey 5-Year Averages retrieved via https://data.census.gov/cedsci on November 2, 2023

**Fig. 1:**
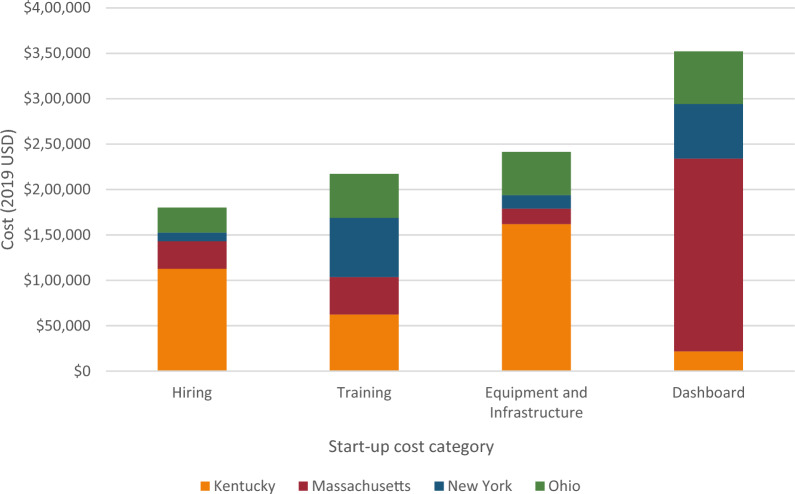
Site contribution of total start-up costs by category

Across all four sites, a total of 45 staff were involved in hiring 71 intervention staff needed to begin implementing the CTH intervention. The mean and median time spent by hiring staff were 771 h and 438 h, respectively. The mean and median cost of hiring across the four intervention sites was $45,018 and $28,992, respectively (Table 2). In total for all four sites, hiring costs were $180,072. The Kentucky site accounted for 63% ($112,602) of this cost, despite using fewer hiring staff (n = 6) than the New York site (n = 25), which accounted for 5% ($9,487) of total hiring costs (Fig. 1). These differences may be attributed to the number of new staff hired. There were 41 staff who were not “new” hires but whose time was reallocated into CTH intervention activities, and therefore did not have hiring costs, but who were trained on the CTH intervention resulting in a total of 112 staff who received training from 84 trainers.

**Table 2:** Hiring and training start-up cost of implementing the communities that HEAL intervention across all sites

	Hours		Reported wage		Using O*NET Wages—State		Using O*NET Wages—National	
	Mean	Median	Mean	Median	Mean	Median	Mean	Median
All Hiring Staff* (N* = *45)*	771	438	$45,018	$28,992	$39,293	$25,181	$43,592	$40,087
All Training (N = 197)	1143	1146	$54,292	$55,361	$68,150	$71,005	$67,620	$61,290
*Trainers (N* = *84)*	230	125	$15,681	$10,714	$21,515	$9,755	$18,502	$17,010
*Trainees (N* = *113)*	913	798	$38,610	$35,631	$46,635	$41,111	$49,118	$44,280
Total hiring and training	1914	1449	$99,310	$75,326	$107,443	$98,112	$111,212	$101,377

All costs reported in 2019 dollars; costs calculated as Total Cost = (∑ Hours* Wage); O*NET is Occupational Information Network (National Center for O*NET Development, 2022). O*NET wages include 34% fringe

Table 1 also reports total training cost by site. CTH intervention staff were trained on community engagement, communications campaign, and the three pillars of the EBP menu: MOUD, OEND, and safer prescribing [9]. Eleven individuals spent time as both trainers and trainees; their time was applied to the total number of hours in both groups, but their trainer and trainee hours were allocated separately (i.e., not duplicated). Trainers spent an average 230 h and median 125 h training the 71 newly hired, and 41 intervention staff whose time was reallocated (Table 2). The average and median cost of trainer time across sites was $15,681 and $10,714 respectively. Most of the trainer time consisted of developing and preparing materials to conduct the training rather than time with the trainees themselves.

The intervention staff across all four sites accumulated an average of 913 h (median of 798 h) being trained in the CTH intervention. The average and median cost associated with staff who received training across the four sites is $38,610 and $35,631 respectively. Massachusetts had the lowest training cost across the four sites, accounting for 19% of the overall training costs for all four sites, compared to the New York site which had the highest training cost, accounting for 30% of the total training costs (Fig. 1).

Table 2 reports average and median labor costs for hiring and training across the four sites using reported wages and O*NET wages at the state and national level. The mean and median cost of labor to prepare for the implementation of the CTH intervention across all four sites using administrative data are $99,310 and $75,326 respectively. Mean costs were 8–12% higher and median costs were 30–35% higher using state or national wage data. The impact of the different wages varied by site, although Ohio was the only site where using standardized O*NET wages resulted in lower total labor costs than using reported salaries and fringe benefits (Additional file 1: Table S3).

Additional file 1: Table S4 summarizes equipment, infrastructure, and dashboard costs by site. The Kentucky site accounted for approximately half of the total equipment cost across the sites, with a cost of $79,552. In Kentucky, equipment costs were identified through invoices and study records and included reimbursed mileage and travel time to and from communities in which the CTH intervention would be implemented, which are captured as “Other” costs. The Kentucky site’s equipment cost also included IT specialist time needed to mount and install equipment in community office space. New York and Massachusetts had the lowest equipment costs of $14,845 and $17,125 respectively, likely because CTH intervention staff were housed in offices that already had some equipment in place for their own staff.

The Kentucky site was alone in incurring new infrastructure costs to prepare for CTH implementation totaling $77,305. These space and infrastructure costs include time spent by administrative staff to select space and costs of executing contracts for new leases. The Kentucky site also paid quarterly leases for the space needed to support the CTH staff in multiple communities. During the start-up period, lease payments totaled $134,180 but are not included in infrastructure estimates to remain consistent with the other sites.

The cost to construct community dashboard portals varied by site due to differences in processes for the portal and dashboard creation. The cost of Kentucky’s dashboard was the lowest at $21,654, which included the time of a computer programmer using open-source software and libraries (free to anyone on the internet). Massachusetts incurred the highest cost at $212,408, which consisted of informatics, data management and programming, website development and hosting, and non-service (hardware and software) costs. The New York and Ohio sites reported similar costs of $60,006 and $57,752, respectively. The New York site contracted out to a university information technology department for development and software. The Ohio site used university personnel proficient in software development to develop the portal, data science engineers to develop dashboards, and system engineers to articulate the infrastructure for hosting.

## Discussion

This cost analysis presents the costs incurred to prepare for the implementation of the CTH intervention in 34 unique communities throughout Kentucky, Massachusetts, New York, and Ohio. These are the first reported initial investments required to implement a large-scale, community-driven approach to address the opioid epidemic. The implementation of evidence-based practices should account for the economic implications of starting a new intervention or approach, which are rarely reported in the economic evaluation literature. Reporting on the investments can inform future communities and policymakers of the resources, time, and costs required to initiate a community-level intervention at this scale. The start-up costs from this analysis may be generalizable to any community-level intervention that follows a coalition-based, data-driven model, as the processes for hiring, training, and infrastructure in CTH likely would be similar regardless of health outcomes targeted. Due to key site-level differences in implementation approach, our analysis also allows stakeholders to view implementation through three distinct staffing models: hospital-academic (Massachusetts), university-academic (Kentucky and Ohio), and community-leveraged (New York).

Both labor and non-labor costs varied widely across research sites. In general, hiring and training staff for the implementation of the CTH intervention were the largest cost components consistently across all sites due to the time commitment required for hiring, gathering and preparation of training materials, conducting training, and the time commitment of the new hires being trained. While the elements of hiring and training labor costs were similar across sites, labor cost estimates varied depending on the breakdown of staff types involved in training and hiring and how the sites staffed the CTH intervention. In Massachusetts, substantially more staff were involved in hiring compared to Kentucky, for example, yet overall hours spent hiring new staff in Massachusetts was substantially less than in Kentucky. This could be explained by the HEAL-specific career fairs hosted by the Massachusetts site, which led to invitations for group interviews/simulations with groups of staff. Additionally, due to their hospital-academic model of implementation, the Massachusetts site was able to reallocate existing staff time in many cases to create a CTH intervention team rather than bring on new hires. On the other hand, the New York site leveraged community resources to lead hiring efforts; health departments and local organizations in each community implementing the CTH intervention led the hiring process independently which led to variability in hiring costs across the individual communities. Overall, 25 different staff in New York were involved in hiring, many more than Kentucky and Massachusetts, but New York communities reported spending substantially less total time hiring than Kentucky or Massachusetts. One possible explanation is that in New York each community hired local individuals who were already known to the health departments due to their work history in the substance use field. Additionally, as New York community sites were not affiliated with a university or a hospital, they may have had a less time-consuming and bureaucratic process for hiring staff. On the other hand, the New York community model incurred higher training costs, which may be a consequence of bringing on non-academic partners to implement an intervention. The hiring and training costs incurred by the New York site may represent more real-world costs to a community replicating the CTH intervention compared to the other sites.

Equipment, infrastructure, and dashboard costs varied widely across the four CTH sites. Kentucky had the highest equipment cost and was the only site reporting new infrastructure purchases and space leasing costs. These costs were incurred for the purpose of intervention staff having their own space in their respective communities, and to reduce staff travel time to complete intervention activities considering the long distance between communities and the University of Kentucky. The extent to which future communities would need to invest in these types of designated spaces may vary; however, Kentucky’s model provides valuable insight for communities with similar space needs. In this case, infrastructure investments were integral to supporting the launch and ongoing activities of CTH. While the other sites did not incur new infrastructure costs, it is important to note the opportunity cost of existing infrastructure and that leveraging existing space means that space is no longer available for other programs. Therefore, stakeholders should consider full organizational needs when determining the cost of new versus existing infrastructure. In Massachusetts, CTH intervention staff had office space within the centralized hospital-academic system from which they traveled to their respective communities. However, the analysis presented in this manuscript only includes travel related to infrastructure preparation and/or training. Future cost analyses of CTH will include travel costs related to implementing the intervention. In comparison to Kentucky, travel times to communities in Massachusetts were shorter. Kentucky, New York, and Ohio had relatively low dashboard and portal costs compared to Massachusetts. This was due to the availability of software developers at academic institutions who were involved in creating the dashboards. Additionally, open-source frameworks, libraries, and software were utilized in the creation of these dashboards, which may contribute to reducing the cost of dashboard creation. In comparison, Massachusetts’ high dashboard costs occurred due to outsourcing the creation of the dashboard and website hosting.

Despite attempts to standardize collection of start-up costs, our findings are limited by the quality and heterogeneity of our data. The start-up of the CTH intervention was not completely standardized across the sites, and therefore processes to prepare for implementation differed across each site depending on whether the site followed a university-academic, hospital-academic, or community-leveraged model. This ranged from how employees were hired and trained, the start-up phase timeline, and some cost data that were not collected consistently by all sites (e.g., travel costs related to infrastructure and space preparation). Interpretation of cost per capita presented in this analysis is limited in that it does not account for OUD prevalence, which would be a more direct measure of cost per target population. Although the target population of CTH is those with OUD, the per capita cost represents the cost burden of those who will pay the start-up costs (i.e., the broader community). Decisionmakers may choose to weigh per capita costs by OUD prevalence to inform spending of public health resources based on the target population. Furthermore, the COVID-19 pandemic interrupted the end of the start-up phase, and it is unclear how the number of staff hired or the training modalities may have been impacted by the pandemic. To capture all relevant start-up costs we extended the start-up data collection phase 4 months into the implementation of the intervention, but we do not include the cost of developing or deploying the CTH intervention in our start-up analysis. The ability to report on four different research sites’ processes for preparing to implement this opioid overdose intervention is a unique contribution to the field and can help stakeholders understand the potential resources involved in a wide-scale community-engaged intervention such as the CTH intervention.

## Conclusion

The variation in start-up cost may be of interest to policymakers when deciding how to initiate implementation of the CTH intervention and other large-scale community-based interventions. Implementation using a community-leveraged model similar to the one used by the New York site may be appealing. Hiring and training labor costs and other costs were the lowest for this site due to existing infrastructure, relationships, and support from local organizations. Kentucky and Ohio followed a more centralized top-down approach to the start-up period, specifically for hiring, which may require that the CTH intervention be driven by an academic institution. The Kentucky site also provides an example of the cost to implement an intervention in communities with little existing infrastructure and may inform start-up in rural communities. We found that in communities where there is existing infrastructure, start-up may be less costly if resources are able to be allocated to the new intervention without burdening other programs. Overall, the modest cost burden of $0.06 to $0.67 per community member demonstrates the feasibility of all four start-up models for a large-scale community-level intervention.

### Supplementary Information

**Additional file 1.** Supplementary material for cost data collection, intervention staff categories, hiring and training costs by site, and other start-up costs by site.

## Data Availability

All data generated or analyzed during this study are included in this published article. Administrative wage data generated and analyzed during this study are not publicly available due to their sensitive nature. However, generalizable wage data can be found on O*NET (www.onetonline.org). Dr. Kathryn E. McCollister (kmcolli@miami.edu) may be contacted for further wage data requests.

## Abbreviations

MOUD: Medications for opioid use disorder
EBP: Evidence based practice
OUD: Opioid use disorder
CTH: Communities that HEAL
HCS: HEALing Communities Study
