# Supplementary material for: Cost of start-up activities to implement a community-level opioid overdose reduction intervention in the HEALing Communities Study
Source: Addict Sci Clin Pract. 2024 Apr 2;19:23. doi: 10.1186/s13722-024-00454-w (PMC10988809; doi:10.1186/s13722-024-00454-w)
Supplement: Supplementary file 1 — Additional file 1. Supplementary material for cost data collection, intervention staff categories, hiring and training costs by site, and other start-up costs by site. [file 13722_2024_454_MOESM1_ESM.docx]

**Additional file Material S1: Start-up cost data collection form to guide interviews**

INSTRUCTIONS

The purpose of this instrument is to understand the costs associated with start-up of phase 0 of the Healing Communities Study (HCS).

These may include costs associated with training, hiring, development of the dashboard/portal, and any other materials or infrastructure involved in start-up of phase 0.

Please report all costs in the Training, Hiring, or Purchasing tab. Dashboard and portal costs should be included in the Purchasing tab.

Please assign staff and facility a unique ID or code so that wages remain private. Names should not be included and the ID should not be based on identifying information (e.g., initials).

NOTE: in the Purchasing tab, please include one-time space costs (e.g., leasing agent fees, renovations). Exclude ongoing costs (e.g., rent, subscription services such as conference call lines and Zoom subscriptions).

| **Hiring of staff to support implementation of HCS during the Start-up Phase of the Study (Start-up of Phase 0)** | | | | | | | | |  | **Notes** |
| --- | --- | --- | --- | --- | --- | --- | --- | --- | --- | --- |
| County name | Activity* | Date | Staff ID | Role/occupation of person who performed activity | Actual wage of person who performed activity | Type of staff being hired | Estimated total hours per staff person hired | Number of staff people hired |  |  |
|  |  |  |  |  |  |  |  |  |  |  |
|  |  |  |  |  |  |  |  |  |  |  |
|  |  |  |  |  |  |  |  |  |  |  |
|  |  |  |  |  |  |  |  |  |  |  |
|  |  |  |  |  |  |  |  |  |  |  |
|  |  |  |  |  |  |  |  |  |  |  |
|  |  |  |  |  |  |  |  |  |  |  |
|  |  |  |  |  |  |  |  |  |  |  |

*Activities may include:

- Creating and posting job descriptions
- Reviewing applicants
- Interviewing
- Hiring decision-making
- General onboarding activities that do not include HCS-specific training

| **Purchasing (not associated with trainings)** | | | | | |
| --- | --- | --- | --- | --- | --- |
| County | Date | Item or service purchased | Number of units purchased | Unit cost | Total Cost |
|  |  |  |  |  |  |
|  |  |  |  |  |  |
|  |  |  |  |  |  |
|  |  |  |  |  |  |
|  |  |  |  |  |  |
|  |  |  |  |  |  |
|  |  |  |  |  |  |
|  |  |  |  |  |  |

| **Resources for Trainings to support HCS Implementation during the Start-up Phase of the Study** | | | | | | | | | | | | | | | | |  |
| --- | --- | --- | --- | --- | --- | --- | --- | --- | --- | --- | --- | --- | --- | --- | --- | --- | --- |
| Training Event Details | | | | | | | | | Attendance Details (list either the attendees themselves or the count in the rows below) | | | | | | | |  |
|  |  |  |  |  |  |  |  |  | Community members (non-HCS staff) | | | HCS Staff receiving training to support Implementation | | | | | Total Attendees |
| Date | Training Topic | Location | Role/ occupation of Trainer/ Training Facilitator | Actual wage of Trainer/ Training Facilitator IF INTERNAL | Cost of Trainer/ Training Facilitator IF EXTERNAL | | Other Costs associated with training event | Length of Training (Hours) | Coalition members | Other Stakeholders | CAB members | | Community Engagement Facilitators | Program Managers | Other HCS Staff assigned to a specific community | Other HCS staff not assigned to a specific community |  |
|  |  |  |  |  |  |  |  |  |  |  |  | |  |  |  |  |  |
|  |  |  |  |  |  |  |  |  |  |  |  | |  |  |  |  |  |
|  |  |  |  |  |  |  |  |  |  |  |  | |  |  |  |  |  |
|  |  |  |  |  |  |  |  |  |  |  |  | |  |  |  |  |  |
|  |  |  |  |  |  |  |  |  |  |  |  | |  |  |  |  |  |
|  |  |  |  |  |  |  |  |  |  |  |  | |  |  |  |  |  |
|  |  |  |  |  |  |  |  |  |  |  |  | |  |  |  |  |  |
|  |  |  |  |  |  |  |  |  |  |  |  | |  |  |  |  |  |

**Additional file Table S2: Categories of intervention staff hired during Communities that HEAL start-up**

| Intervention Staff Category | O*NET Classification | Description |
| --- | --- | --- |
| Community Engagement/ Implementation Facilitator  (n=27) | Medical and Health Services Managers | - Key to expanding the use of evidence-based interventions within partnering organizations. Work with local change teams to develop implementation blueprints and then facilitate change team meetings that are focused on how their services and/or resources can be expanded to serve more people. - Building a network of community partners and local change teams in identified areas. - Sharing and promoting best practices and knowledge between local community partners, local change teams and researchers. |
| Community Coordinator  (n=14) | Medical and Health Services Managers | - Provides all local supervision and project management oversight. Provides direction to CTH teams composed of Prevention Specialists, Peer Prevention Specialists, Social Workers, and Nurse Navigators. - Engages local coalitions and other local community leaders to participate in and otherwise support the initiative. - Effectively communicates information from leadership to community implementation teams and vice-versa. - Develops day-to-day operations and allocation of resources (i.e, staffing, budget, space) for the community implementation team. |
| Data Coordinator  (n=16) | Clinical Data Managers | - Participates in county meetings and facilitates county in making data-driven decisions. - Collects data on opioid overdose deaths and non-fatal opioid overdose, number and percent of people screened for opioid use disorder, linked to medication treatment for opioid use disorder from county records, provider agencies, and county databases. - Works with data from the Department of Health to improve surveillance on opioid use related indicators. - Provides feedback on data points, functioning and usability of data dashboard and design. |
| Prevention Specialists  (n=6) | Mental Health and Substance Abuse Social Workers | - Conducts overdose prevention and treatment referral within local criminal justice and community settings. - Provides public health counseling to interested clients and conducts overdose prevention training to individuals seeking education and distribution of Narcan kits. - Assist clients who are interested in treatment, including medications for opioid use disorder (MOUD), by linking clients with the CTH Care Navigator. - Engage and support clients with problem solving, coordination and promotion of local services/resources, and assist in stigma reduction through training and educational efforts. - Advocates for the needs and desires of clients by engaging local providers from mental health services, addiction services, and physical medicine to meet the needs of clients. |
| Education/ Technical Assistance Coordinator  (n=4) | Health Education Specialists | - - - - Provides coordination of community trainings such as buprenorphine waiver trainings and weekly overdose prevention didactics. |
| Academic Detailing Pharmacist  (n=2) | Pharmacists | - Perform outreach education that combines direct social marketing traditionally used by pharmaceutical representatives with unbiased content summarizing the best evidence available |
| Treatment Coordinator  (n=2) | Health Education Specialists | - Coordinates outreach and establishment of community treatment networks during planning and implementation. |

**Additional file Table S3: Hiring and training start-up cost of implementing the Communities that HEAL intervention by site for reported wage costs compared to average O*NET state and national wages**

|  | **KY** | | | **MA** | | | **NY** | | | **OH** | | |
| --- | --- | --- | --- | --- | --- | --- | --- | --- | --- | --- | --- | --- |
|  | Reported Wage | Average O*NET  state wage | Average O*NET national wage | Reported Wage | Average O*NET  state wage | Average O*NET national wage | Reported Wage | Average O*NET state wage | Average O*NET national wage | Reported Wage | Average O*NET  state wage | Average O*NET national wage |
| All Hiring *Staff (N=45)* | $ 112,602 | $ 94,279 | $ 116,124 | $ 30,513 | $ 31,882 | $ 28,159 | $ 9,487 | $ 12,533 | $ 10,350 | $ 27,471 | $ 18,479 | $ 19,733 |
| All Training (N=197) | $ 62,260 | $ 80,535 | $ 100,166 | $ 41,212 | $ 61,476 | $ 53,817 | $ 65,233 | $ 90,333 | $ 74,152 | $ 48,461 | $ 40,257 | $ 42,345 |
| *Trainers (N=84)* | $ 7,198 | $ 5,942 | $ 7,728 | $ 13,095 | $ 12,515 | $ 11,398 | $ 34,100 | $ 60,609 | $ 47,688 | $ 8,333 | $ 6,996 | $ 7,192 |
| *Trainees (N=113)* | $55,062 | $ 74,593 | $ 92,437 | $ 28,117 | $ 48,961 | $ 42,419 | $ 31,133 | $ 29,724 | $ 26,464 | $ 40,128 | $ 33,262 | $ 35,153 |
| **Total Hiring & Training** | $ 174,862 | $ 174,814 | $ 216,290 | $ 71,725 | $ 93,358 | $ 81,976 | $ 74,720 | $ 102,866 | $ 84,502 | $ 75,931 | $ 58,737 | $ 62,078 |

All costs reported in 2019 dollars; costs calculated as Total Cost = (∑Hours * Wage); O*NET is Occupational Information Network (National Center for O*NET Development, 2022). O*NET wages include 34% fringe

**Additional file Table S4: Equipment, infrastructure, and dashboard creation start-up cost of implementing the Communities that HEAL intervention across all sites**

|  | **KY** |  | **MA** |  | **NY** |  | **OH** |  | **Mean** | **Median** |
| --- | --- | --- | --- | --- | --- | --- | --- | --- | --- | --- |
| Equipment | $ 79,522 |  | $ 17,126 |  | $ 14,845 |  | $ 47,571 |  | $ 39,766 | $ 32,349 |
| Infrastructure | $ 77,305 |  | - |  | - |  | - |  | $ 19,326 | - |
| Dashboard | $ 21,654 |  | $ 212,408 |  | $ 60,006 |  | $57,752 |  | $ 87,955 | $ 58,879 |
| Other* | $ 5,061 |  | - |  | - |  | - |  | $ 1,265 | - |
| **Total** | $ 183,542 |  | $ 229,534 |  | $ 74,851 |  | $ 105,323 |  | $ 148,312 | $ 91,228 |

All costs reported in 2019 dollars

*****Travel expenses, project management services, and review of RFPs for all of Kentucky infrastructure sites.
